# Medicine in history and history in medicine: the inaugural Davis Coakley Memorial Lecture

**DOI:** 10.1007/s11845-023-03495-3

**Published:** 2023-08-19

**Authors:** Brendan D. Kelly

**Affiliations:** Department of Psychiatry, Trinity College Dublin, Trinity Centre for Health Sciences, Tallaght University Hospital, Tallaght, Dublin 24, D24 NR0A Ireland

**Keywords:** COVID-19, Historiography, History of medicine, Ireland, Sir William Wilde

## Abstract

Professor Davis Coakley (1946–2022) was an outstanding physician, historian, and leader of reform in medical services and education. This inaugural Davis Coakley Memorial Lecture, delivered in The Edward Worth Library at Dr Steevens’ Hospital, Dublin, Ireland, on 28 September 2023, focuses on ‘Medicine in History & History in Medicine’. It explores the position of the physician-historian in medical historiography, discusses Coakley’s extensive historical work (especially his many books about the history of medicine), and concludes with comments about one of Coakley’s great interests: the work of Sir William Wilde (1815–1876). Sir William was a prominent Victorian doctor, father of playwright Oscar, and remarkably prescient commentator on public health in Ireland. Sir William’s comments about patterns of epidemics are especially arresting and relevant today in the immediate wake of COVID-19. Coakley’s interest in Sir William echoed Coakley’s broader commitment to medical care, progressive education, and genuine scholarship that shed light on suffering, illness, healing, and recovery. The fields of both medicine and history are greatly enriched by Coakley’s life and work.

It is a great honour and a real joy to deliver this inaugural Davis Coakley Memorial Lecture on the subject of ‘Medicine in History & History in Medicine’ in The Edward Worth Library at Dr Steevens’ Hospital, Dublin, Ireland, on 28 September 2023. I will start by exploring the position of the physician-historian in medical historiography, discuss some of Coakley’s historical work (especially his books), and conclude with comments about one of Coakley’s great interests, the work of Sir William Wilde (1815–1876). Sir William was a Victorian doctor, father of playwright Oscar, and remarkably prescient commentator on public health in Ireland. But, first, the position of the physician-historian in medical history today, and Coakley’s contribution.

## Physicians, historians, and physician-historians

In 1980, the *Journal of the History of Medicine and Allied Sciences* published an editorial titled ‘Medical history without medicine’ [1]. The weary-sounding editorial writer stated that, in the decades since 1945, the number of professional medical historians had grown considerably, with many primarily trained in history rather than medicine or science. The editorialist saw value in this development, but said that it tended to isolate scholars from practical problems, limit their inquiries to social history, and result in the neglect of clinical medicine, biology, and science. The upshot, they wrote, was an incomplete and sometimes distorted version of history:

*The social history of attitudes toward disease and doctors is a valid and fascinating subject of inquiry, but it does not comprehend the whole of the history of medicine […] it is medical history without basic medical sciences and clinical methods and concepts; that is, it is medical history without medicine.* [1] (p. 7)

This long-standing tension reflected the transition from an era when much medical history was written by medical doctors and little by historians, to a situation where much medical history is written by historians and little by medical doctors. To a considerable extent, this shift makes sense, not least because there is a law against historians practicing medicine, but no law against medical doctors writing history.

Prior to 1980, the accepted historiographical narrative was that trained historians brought objective, systematic historical analysis to the field of medical history, replacing medical doctors’ earlier ‘Whiggish’ accounts of the story of medicine as one of inevitable progress and improvement, which was largely attributable to them. Predictably, perhaps, it soon emerged that this division was nowhere near as crisp as initially presented, and the situation was not nearly as dispiriting as our anonymous editorialist felt in 1980. In 1994, Roy Porter and Mark Micale warned sternly against such a binary distinction in the context of the history of psychiatry in particular:

*The Great Revision of the last three decades pits ‘amateur’ physician-cum-historians writing a naïve and self-serving internalist intellectual history of psychiatry against ‘professional’ historians producing a more methodologically sophisticated, empirically substantial, and sociologically oriented scholarship. But an unbiased examination reveals this idea as a simple misreading of the historiographical record.* [2] (p. 8)

In essence, it turned out that historians knew a lot more medicine than they were given credit for, and physicians knew more history and had deeper critical awareness than was often imagined. As ever, an apparently binary division was both inaccurate and unhelpful.

In addition, other perspectives garnered the attention they merited at around this time, in addition to physicians, historians, and physician-historians. These included the views of patients [3], their families, different staff across health services, and many others. In 1999, John Burnham reflected this in *Health and History*, when he wrote that, by ‘the end of the twentieth century […] historians of medicine needed all the help they could get, from every quarter. The proper focus […] was not an *ad hominem* argument (regarding the formal qualification of the writer), but the quality of his or her contribution’ [4] (p. 273).

In the Irish context, historian Catherine Cox noted, in 2013, that, with a diversity of voices emerging from different backgrounds, ‘an overly hasty dismissal of this scholarship, which was informed by a discrete expertise, will result in the loss of specific medical knowledge and experience, which can bring added depth to a complex and varied subject’ [5] (pp. 345–346). Amidst this diversity of voices, it is necessary and wise to evaluate by output rather than discipline, by quality rather than theoretical framework, and by contribution rather than ideology. We need an awareness of all these factors, but with a focus on validity and insight.

The result is a medical history that is now more critical, inclusive, complex, and sophisticated, especially with trained historians casting their specialist eyes over the entire enterprise. In 2005, Burnham identified five key ‘dramas’ in the history of medicine, relating to the histories of ‘the healer’, ‘the sick person’, ‘diseases’, ‘discovering and communicating knowledge’, and ‘medicine and health interacting with society’ [6] (p. 9). With these themes or ‘dramas’ in mind, and with a broad multiplicity of voices established, the history of medicine finds itself in a refreshed and exciting place, recognising the historical, sociological, and medical dimensions of the stories of medicine, health, and healthcare, in the clinic and in society. This is a history that became ever more arresting in recent years, with the arrival of COVID-19, the emergence of vaccines, and the world left reeling in its wake. We will return to COVID-19 later, after turning our attention to Coakley and his work.

## Professor Davis Coakley (1946–2022): physician, historian, and physician-historian

For now, with the position of the physician-historian more clearly articulated, it is appropriate to turn our focus to Professor Davis Coakley, to mark our sadness at his death in September 2022, to share our joy at having known him, and to express gratitude for his achievements [7]. The loss to Irish medicine and history is profound, the only consolation being the enduring legacy of Coakley’s contributions to his profession, to medical history, and to Irish society over the course of his remarkable career. We celebrate this and explore his historical work today.

First, it is impossible, and would be inappropriate, to remember Coakley without listing a long succession of accomplishments. Coakley steered the establishment of the Mercers Institute for Successful Ageing on the grounds of St James’s Hospital in Dublin. He twice served as Dean of Health Sciences at Trinity College Dublin. His 2014 account of the medical school, *Medicine in Trinity College Dublin: An Illustrated History* [8], is the definitive account, described by Aidan Collins as an ‘excellent and beautiful book’ [9] (p. 133) and by Muiris Houston as ‘a must read for TCD graduates and historians’ [10] (p. 17). Overall, Coakley wrote some 18 books, many concerned with the history of medicine.

Coakley helped shape programmes in academic nursing, occupational therapy, speech therapy, and the medical humanities at Trinity. His impact on the university was enormous, and his work continues to shape the institution today. Coakley’s contributions were substantial, generous, and fuelled by deep-seated passion for medicine, healing, research, and learning. He did a great deal to establish geriatric medicine in Ireland and helped shift perceptions of medicine and medical education in positive, constructive directions over several decades. For these and many other reasons, we are in his debt.

Coakley had several other roles. From 1991 to 1994, he served as Dun’s Librarian at the Royal College of Physicians of Ireland, a post that originated in the 1780s, when one of the College Fellows was appointed to look after the library of Sir Patrick Dun (1642–1713), an eminent physician and former President of the College, who bequeathed his personal library to the establishment. Coakley brought enthusiasm and erudition to the role. I now hold this position with, I feel, as much enthusiasm as Coakley but considerably less erudition.

Coakley’s academic standards and literary skills were apparent across his work on the history of medicine. His 1988 book, *The Irish School of Medicine: Outstanding Practitioners of the 19th Century* [11], was described by David Nowlan in *The Irish Times* as ‘written clearly and compellingly in a narrative style that makes it not only a record of the period and the personalities but also a very good read’ [12]. Coakley’s other historical books include *Doctor Steevens’ Hospital: A Brief History* [13], *Robert Graves: Evangelist of Clinical Medicine* [14], and, with Mary Coakley, *The History and Heritage of St James’s Hospital, Dublin* [15, 16].

*Irish Masters of Medicine*, published by Town House in 1992, was, perhaps, one of Coakley’s finest books [17]. In this magisterial work, Coakley presented engaging and informative biographies of 42 Irish medical personalities, including Abraham Colles (1773–1843), John Cheyne (1777–1836), Robert Graves (1796–1853), Dominic Corrigan (1802–1880), William Stokes (1804–1877), and William Wilde (1815–1876). This book has stood the test of time and remains an essential resource for historians of medicine today. Coakley’s thoughts about Sir William Wilde have, perhaps, special relevance in the era of COVID-19 and merit particular attention today.

## Sir William Wilde (1815–1876): Victorian doctor

Coakley had a long-standing interest in Sir William Wilde, a remarkable Victorian doctor and father of playwright Oscar. In 1994, Coakley’s book *Oscar Wilde: The Importance of Being Irish* explored Oscar’s Irish upbringing and its impact on his development [18]. Jerusha McCormack reviewed the book in *The Irish Times* and noted that ‘hitherto biographers passed lightly over the fact that Oscar Wilde was born and educated in Ireland. Coakley makes those first twenty years the centre of an entire magnetic field’ [19]. Following ‘Coakley’s research on the Merrion Square milieu of Wilde’s time […] much of Wilde’s work will resonate with a difference’. Coakley was also involved in preserving Oscar Wilde’s birthplace, 21 Westland Row, now a creative writing centre in Trinity.

Coakley had a particular interest in Oscar’s father, Sir William, whom Coakley described as ‘one of a group of remarkable physicians and surgeons who put Dublin medicine on the centre stage of international medicine’ in the nineteenth century [18] (p. 1). Sir William was, at times, a controversial figure, but his contributions were many, ranging from practicing ophthalmology to researching Irish antiquities, from analysing census data to exploring Irish history, heritage, and folklore with sympathy, insight, and critical rigour [20].

Sir William wrote at particular length about plagues, epidemics, pestilences, and various other disasters in Irish history, based, in part, on Irish census data, liberally mixed with Sir William’s knowledge of history and folklore. The results of the 1851 census formed the basis of much of this work and were published in ten volumes totalling 4533 pages, of which Sir William single-handedly wrote two volumes, Parts III and V [21]. Sir William presented the latter in two volumes, collectively called ‘Tables of Deaths’. The grim title conceals a multitude of wonders. The first volume of Part V amounts to 560 pages and includes a 270-page ‘Table of Cosmical Phenomena, Epizootics, Famines, and Pestilences in Ireland’ [22]. It is an historical treasure trove [23].

Sir William starts his epic account in the ‘Pagan or Pre-Christian Period’ and recounts a bewildering array of illnesses, misfortunes, and bizarre celestial events that afflicted Ireland up to 1851. The first entry in his Table recorded the deaths of 5000 men and 4000 women in one week in ‘the place now called Tallaght, near Dublin’ owing to ‘some sudden epidemic’ which was ‘the first recorded pestilence in Ireland’ in approximately 2680 BC [22] (p. 41). The precise nature of this epidemic remains obscure today. More was to follow: ‘a great epidemic pestilence’ arrived in AD 806 and ‘the moon was turned into blood’ (p. 59). In AD 875, there was ‘great wind, lightening, and thunder’, and ‘showers of blood were afterwards shed, so that lumps of gore and blood were visible’ (p. 60).

Sir William sourced much of this information from other historical accounts, including the Annals of Ulster, which recorded events such as ‘a great Leprosy’ and ‘running of blood’ in AD 950 (p. 63). Sir William felt that this was an especially significant report:

*These two notices would appear to apply to Syphilis, (and if so, to fix the introduction of it into Ireland), and the circumstances of the period tend to favour that idea. For some years before, there had been several Danish invasions, and immediately preceding the year 950 the Danes plundered a great part of Leinster, and took many captives - in one instance ‘upwards of three thousand persons’, so that the accession and spread of venereal affections is likely to have occurred at the time referred to.*

But it is, perhaps, Sir William’s comments about epidemics that are the most arresting and relevant today in the immediate wake of the COVID-19 pandemic [24]. In 1856, Sir William wrote:

*Many of the plagues from which this country suffered were continuations of those great waves of pestilence which had already passed (according to the general course of plagues) from the East, over the European continent, frequently carried along the track of human intercourse, by commercial dealings, or borne onward by hostile navies or invading armies; but others were more localised, were of domestic growth, and had their birth, and expended themselves within the circuit of this island - seldom spreading beyond its limits.* [22] (p. 2)

With this level of prescience and insight, it is little wonder that Coakley had a long-standing interest in Sir William and his work. Of the many (but not nearly enough) conversations I had with Coakley, perhaps the clearest in my memory is one of the briefest, when I bumped into him in Hodges Figgis book shop on Dawson Street in Dublin some years ago [7]. I was lingering in the history section, looking at a book about Oscar Wilde. Coakley spotted me, and we got to discussing Sir William’s contribution to Irish medicine. When I mentioned my admiration for Coakley’s book about the Wildes, Coakley said he was thinking about a new edition—something that would have been enormously valuable to anyone interested in Sir William. Sadly, it was not to be.

## Conclusions

Coakley made many contributions to both medicine and history. For me, his greatest contributions as an educator were his demeanour and approach: gentle, erudite, compassionate, and very effective. Few have contributed so much, to so many, for so long, and with such humility. The Coakley that I remember best is the kind, soft-spoken man who fostered my early interest in the history of medicine, writing to me saying that it was ‘very encouraging’ to see people ‘publishing on medical history and keeping up the tradition’ [7]. When we met over subsequent years, Coakley never failed to tell me something new, interesting, and unexpected about the history of medicine.

At the end of his Introduction to *Irish Masters of Medicine*, Coakley wrote that he hoped that the accomplishments of the doctors he discussed would ‘emphasise the importance of creating and supporting an ethos of research within our medical schools and hospitals and that the biographies may inspire medical students and young Irish doctors to face the challenges posed by the major health issues of our own time’ [17] (p. 6). The same might be said of Coakley—a modern master of medicine who enriched both medicine and history through his life and work.
